# Gender differences in mortality and risk factors in a 13-year cohort study of street-recruited injecting drug users

**DOI:** 10.1186/1471-2458-14-440

**Published:** 2014-05-10

**Authors:** Linn Gjersing, Anne Line Bretteville-Jensen

**Affiliations:** 1 Norwegian Institute for Alcohol and Drug Research (SIRUS), PB 565 Sentrum, Oslo 0105, Norway

**Keywords:** Drug user, Injecting drug user, Needle exchange programmes, Overdose, Mortality, Cohort study, Data linkage

## Abstract

**Background:**

Injecting drug users (IDUs) are at risk of premature mortality. This study examined gender differences in mortality, risk factors, and causes of death among IDUs.

**Methods:**

In a 13-year cohort study including 172 street-recruited IDUs from Oslo, Norway in 1997, interview data was merged with the National Cause of Death Registry. Crude mortality rate (CMR) and indirect standardized mortality ratio (SMR) were estimated with 95% confidence intervals (CI). A log-logistic multivariate survival analysis model was estimated for the full sample. For a smaller data set (1.1.1998-31.12.2004) the influence of substitution treatment and prison were assessed using cox regression survival analysis.

**Results:**

Eight females and 37 males died. Acute intoxications were the most common cause of death. Women were more at risk in the short-term, but more protected in the long-term. CMR was 16.0 [95% CI 8.0, 31.9] for women and 26.0 [95% CI 18.0, 35.8]) for men. SMR was 39.4 [95% CI 0.2, 220.8]) for women and 21.3 [95% CI 5.7, 54.1] for men. More women injected heroin (98% vs. 88% [*x*^2^ = 3.5, p = 0.063]), used prescription drugs (73% vs. 52% [*x*^2^ = 5.6, p = 0.018]) and combined these to inject (45% vs. 26% [*x*^2^ = 5.9, p = 0.015]). Mixing prescription drugs in heroin injections, and sex work (only women) were associated with decreased survival time. There were no gender differences in access to substitution treatment, while significantly more men had been in prison (74% vs. 51% [*x*^2^ = 7.5, p = 0.006]). The instance of substitution treatment and prison significantly decreased the mortality risk. Prison release increased the risk, but not statistically significantly.

**Conclusions:**

There were gender differences in mortality and risk factors; sex work and prison were gender specific risk factors. These factors should be investigated further to better design future preventive measures.

## Background

Drug users have a risk of premature mortality 10 to 20 times higher than the general population [1]. Fatal overdose is the most common cause of death among drug users [2-4]. However, this population also has an elevated risk of death from suicide, violence, transport accidents and hepatitis C-related causes [5-7]. This suggests that they are exposed to a wide range of risk factors. These risk factors differ between men and women [8-10]. A better understanding of these differences could provide new insights into how deaths may be prevented.

The risk of premature mortality is higher among male injecting drug users (IDUs) than females [8-10]. Garrick and colleagues [11] found a male to female ratio of five to one in heroin-related deaths. Gossop and colleagues [12] found a ratio of three men to one woman in a cohort study, yet when number of deaths was assessed at follow-up they found a ratio of seven men to one woman. Many studies report a higher crude mortality rate (CMR) for male IDUs compared to female IDUs [13-15], and the majority of these studies also find that women have a higher standardized mortality ratio (SMR) than men [16-18]. The interpretation of CMR and SMR is described in the Methods section of this paper. The gender difference in SMR reflects the gender differences in mortality of the general population.

Despite the higher risk of mortality among male IDUs, some risk factors seem more unique to women. Female IDUs appear to use and inject drugs as frequently or more than men [19-23]. Additionally, women are more likely to engage in sex-work than men [24-27], which has been associated with premature mortality [27]. Possibly the pattern of substance use by females, in addition to their engagement in sex-work increases their risk of mortality.

On the other hand, female drug users are found to seek treatment earlier in their drug career and they are less likely to relapse [28,29]. Homelessness and shelter use have been associated with increased mortality risk [30-32], and there are more men than women in this population [31,33,34]. Moreover, the first weeks after prison release are associated with increased mortality risk [35-37]. Women are less likely to be incarcerated [24,25,37], and less likely to re-offend [38,39].

Thus, there appears to be some risk factors specific to women but not to men, and vice versa. The association of these gender differences with short- and long-term mortality is assessed in this cohort study of street-recruited Norwegian IDUs followed from 1997 until 2010. Norway is a Nordic country with approximately 5 million inhabitants and the capital Oslo has 600,000 inhabitants [40]. There is an estimated 8,700 to 12,300 IDUs (predominantly opioids) and approximately 3,000 of them live in the capital [41]. Since 1997, Norway has had one of the highest rates of IDUs among problem drug users in Europe [1]. In 1997, 1.5 million syringes were distributed and 103,000 individual visits were registered in the only needle exchange programme (NEP) in Oslo. This illustrates the high prevalence of injection use in the city.

Furthermore, Norway has one of the highest reported drug-induced mortality rates in Europe [1]. There are between 250 and 300 drug-induced deaths annually and about one-third occurs in Oslo [42]. Oslo is therefore a suitable setting for an examination of mortality and associated risk factors. The aim of this study was to assess gender differences in mortality, risk factors and causes of death. This study includes an out-of-treatment population of IDUs whom reported severe risky drug use behaviour at the time of baseline interview. The study is also likely to include IDUs who would not have been available for inclusion in studies where participants were recruited from treatment or prison settings. The findings may therefore provide new insights into how these deaths may be prevented.

## Methods

### Design and study setting

This was a prospective cohort study among street-recruited IDUs outside the only NEP facility in Oslo in March, June and September 1997. This was the only facility where IDUs could obtain clean injecting equipment for free in Oslo at the time. The NEP facility was a bus that was situated in different locations in the city centre throughout the evening. The city centre is fairly small and it was rarely more than ten minutes walking distance between the different locations. Therefore subjects were recruited at various locations in the city centre.

Interview data was merged with the National Cause of Death Registry between 1997 and 2010 using the subjects’ social security number. Intake and discharge dates for opioid substitution treatment (OST) were obtained from the OST programme in Oslo between 1.1.1998 and 31.12.2004. The Norwegian OST programme was established January 1^st^ 1998 [43] and therefore it was not possible to obtain intake dates prior to this. Incarceration dates and release dates between 1.1.1997 and 31.12.2004 were obtained from Norwegian Correctional Services.

### Participants, recruitment and interviews

Participants were recruited outside the NEP facility after they had collected injecting equipment. Researchers and trained research assistants from The Norwegian Institute for Alcohol and Drug Research recruited and interviewed the participants. The researchers and trained research assistants were situated on the street outside the NEP and they operated separately from the facility. This means that NEP staff were informed that the study was conducted outside their bus, but they were not involved in the recruitment process nor in any other aspect of the study.

The inclusion criterion was for people to have injected at least once in the previous four weeks. Each interview took approximately 15 minutes to complete and was conducted out of earshot from others. No monetary incentives were given for participation.

### Representativeness

The NEP facility was the primary source for clean needle and syringes in 1997. This means that most IDUs in Oslo would have been likely to visit the facility at some time during the year. The data was collected three times over the year (March, June and September) which increased the likelihood of obtaining a representative sample. Furthermore, the gender and age distribution of our sample was similar to what was recorded for IDUs in Norway at the time [44]. People who inject drugs regularly are more likely to attend the NEP than those who inject less frequently. Consequently our sample probably included a higher proportion of the former population than the latter.

We have no information about those who refused to participate in the baseline interview. However, from the 286 subjects who were invited to participate, 172 agreed to participate in the long-term follow up study by providing their name and social security number. Those who did not agree to long-term participation answered the questionnaire anonymously. Those who agreed to participate did not differ from those who did not (n = 114). Both groups had a similar distribution in terms of age, gender, education, age at first injection, income, amount of heroin per injection and total amount of heroin consumed [45].

### Measures

The study questionnaire comprised detailed questions about age, gender, education, current living situation, and sources of income (work, social benefits, dealing, theft and sex work) and amount of income from each income source. In this study dealing and theft were defined as illegal activities. The questionnaire also included questions about alcohol; the amount and frequency of cannabis use; the frequency of cocaine, LSD and ecstasy use. In addition, the respondents were asked if they had used heroin and if they had, their mode of intake (by injections, inhalation or smoking) and the amount of heroin in their last injection (if they had injected). The respondents were also asked about the age of their first injection, injection frequency and what substance they most commonly injected: heroin, amphetamine, both or other substances.

Furthermore, the questionnaire comprised questions about prescription drugs (frequency, type of drug and amount). In 1997, methadone and buprenorphine were not available as prescription drugs in Norway. Instead prescription drugs such as pain medication, sedatives, hypnotics and antiepileptic drugs were available. The respondents were also asked if they mixed prescription drugs and heroin and if they did, how often and what quantity of prescription drugs were used. The questionnaire is described in more detail elsewhere [45].

The National Cause of Death Registry provided the dates of death and the primary causes of death of the participants. Causes of death were categorized by Statistics Norway according to the international classification system (ICD-9 codes). We divided the primary causes of death into six categories. The first category was acute intoxications with three subcategories “due to use of opioids (F11.0)”, “due to use of sedatives or hypnotics (F13.0)” and “Accidental poisoning by and exposure to narcotics and psychodysleptics [hallucinogens], not elsewhere classified (X42.0)”. The other categories were dependence syndrome (F11.2, F19.2), suicide (X70.0, X71.9), acute infections (A39.8, A41.9), chronic infections (B18.2, B20.7, B24.0), and other causes (C49.6, J45.9, K70.3, R99.8, V48.6, W74.8, X59.9 X99.8, Y21.8).

The OST programme in Oslo provided intake and discharge data, and The Norwegian Correctional Services provided incarceration dates and release dates. The information from the OST programme and The Norwegian Correctional Services were used in sub-analyses in a smaller dataset.

### Data linkage

Staff at the Norwegian Social Science Data Services performed linkage of data using mortality data from The National Cause of Death Registry and OST dates for intake, discharge, incarceration and release. The social security number was used for matching purposes. A linked data set was then provided to the researchers.

### Variables and data analyses

Data analyses were completed using Stata version 13.0 [46]. Chi square tests were used for the assessment of differences in baseline characteristics between genders. The cut-off points for the dummy variables “age” and “length of injection career” were based upon Darke and colleagues paper from 2011 [3]. The cut-off point for “Total monthly income” was the median value for the total sample which was 33,000 Norwegian Kroner (NKR). This was approximately 3,560 Great British Pounds (GBP). The cut-off for the total monthly amount of heroin was the median amount of heroin used by the total sample (12.9 grams).

Crude mortality rate (CMR) was calculated by summing the person years (PY) contributed by each participant, by gender and calendar year, then summing the number of deaths by the same groups and calculating a rate per 1000 PY. Indirect standardized mortality ratio (SMR) was calculated by dividing the observed deaths in the cohort by the expected deaths if the cohort had the same specific rates as the death rate in the standard population. The standard population was the general population in Norway between 1997 and 2010 based on age and gender specific rates [40]. The SMR was calculated using the age groups 15–19, 20–24, 25–29, 30–34, 35–39, 40–44, 45–49 and 50–54 years. All rates and ratios were reported with 95% Confidence Intervals (CI).

Two types of survival analyses were conducted. In the first analyses, time-at-risk was the period between the date of baseline interview (interviews conducted March, June or September 1997) and December 31^st^ 2010. A continuous time model could thus be used. Incomplete spells were right-censored. The proportionality assumption for gender was not satisfied and therefore a proportional hazard model such as a Cox regression model could not be used. An Accelerated Failure Time (AFT) parametric model was used instead.

AFT models use log (time-to-failure), rather than risk (hazard) of failure [47]. The regression coefficient B^*^_k_ in AFT models summarizes the proportional effect on survival time T to a unit change in the corresponding covariate [47]. However, it is more common to present the exponentiated regression coefficients, which are called time ratios (TR) [47]. Therefore, this paper reports TR for each covariate’s estimates. TR ranges between 0 and infinity and a coefficient above 1 implies longer duration of survival, while a coefficient below 1 implies shorter duration [47]. For example, if TR for men in a mortality study is three, it means that men have three times longer survival then women. On the other hand if TR for men is 0.3, then men have 70% shorter survival then women.

The three AFT models “Log-Logistic”, “Log-Normal” and “Weibull AFT” were assessed [47]. The Log-Logistic model was chosen based upon an assessment of the AIC criterion and Log-Likelihood estimates.

Unobserved heterogeneity (“frailty”) was controlled for by estimating the models using a Gamma specification [47]. To check robustness, the same assessment was conducted with Log-Normal and Weibull AFT models.

The multivariate Log-Logistic model was theoretically based and we used known risk factors for increased mortality among drug users. These factors were age, sex work, length of injecting career, injection frequency, combination of heroin and prescription drugs in injections and alcohol use [9,12,13,17,18,27,48-50].

Almost all participants (90%) had injected daily or almost daily and therefore there was not enough variation in frequency of use to include this variable in the regression analyses. Since we adjusted for the combination of prescription drugs in heroin injections, we could not use “Any use of prescription drugs” or “heroin use” as separate variables.

There were no reports of income from sex work from men, while 21 of the 44 women did. Therefore sex work was only included as an independent variable in Model 3, where females were analysed separately from males. In model 4, males were analysed separately, excluding sex work from the model. It was not possible to control for frailty in model 3 and 4, which was most likely due to a small sample size.

In the second survival analysis, time-at-risk was the period between 1.1.1998 and 31.12.2004. “Total years in OST” was used as the variable for substitution treatment. “Total years in prison” was used as the variable for incarceration. “Prison release” was included in the model as a time-dependent covariate. Since data on imprisonment and prison release was available from 1.1.1997, data was left censored (imprisonment dates before 1.1.1998 were omitted from the analyses). Incomplete spells from 31.12.2004 were right censored. Data had to be split into incarceration episodes. Due to the organisation of data, it was not possible to use the Log-Logistic model. However, in this limited time model the proportionality assumption was satisfied also for gender. The proportionality assumptions were tested using Schoenfeld residuals, and scaled Schoenfeld residuals [51]. A Cox regression survival model could therefore be applied and hazard ratios (HR) and 95% CI were reported.

Four models were assessed. Model 1 comprised of “total years in OST”, “total years in prison” and “prison release”, to measure the effect on mortality without the other variables. “Prison release” was the risk up until three weeks after release. In model 2, the original variables were added to the analyses. In model 3, women were analysed separately and in model 4, men were analysed separately. It was not possible to assess for unshared frailty (unobserved heterogeneity) in Cox regression analyses [52] and consequently this was not assessed.

The differences in baseline characteristics between the women who reported income from sex work and those who did not, were assessed in post-hoc analysis. The reason for the post-hoc analysis was that sex work significantly decreased survival time in the Log-Logistic analyses and we wanted to determine possible reasons for this. One hypothesis was that high income from sex work allowed these women to consume more drugs and thereby shortening their survival time. To explore this further, we compared the women who reported income from sex work to women who did not. Fisher’s Exact Test was used. For the continuous variables of “total monthly income”, “number of prescription drugs used yesterday”, “amount of heroin per injection”, “number of days used heroin in the past month” and “total amount of heroin used in the past month” a two-sample *t*-test with equal variances was used.

For all analyses, the significance level was set at 5% level, unless otherwise stated in the text.

### Ethics

This study was approved by the Norwegian Medical Ethics committee, the Norwegian Data Inspectorate and the Norwegian Board of Health Supervision.

## Results

### Characteristics of the cohort

Our cohort of 172 IDUs, was made up of 44 women and 128 men. The mean age at the baseline interview was 32.5 years (sd 7.1). Women were on average five years younger than the males (28.9 vs. 33.7). Table 1 shows that there were no gender differences in educational attainment, but there were significant gender differences in sources of income. Only 2% of the 44 women reported income from work compared to 20% of the 128 men (*x*^2^ = 7.6, p = 0.006). In comparison, 48% of the women reported sex work as an income source, whereas no men reported this as an income source (*x*^2^ = 69.6, p < 0.001). More women than men reported a total monthly income above the median monthly income in the total sample which was 33,000 NKR (61% vs. 45% *x*^2^ = 3.7 p = 0.054).

**Table 1:** Baseline characteristics amongst the 172 IDUs

**Characteristics**	**Females**	**Males**	***x***^**2**^**, p-value**	**Total**
	**n = 44 (100%)**	**n = 128 (100%)**		**n = 172**
Age groups				
≥ 30 years	22 (50%)	85 (66%)	*x*^2^ = 3.8 p = 0.053*	107 (62%)
> Mandatory years of education^a^				
Yes	29 (66%)	94 (73%)	*x*^2^ = 0.9 p = 0.340	123 (72%)
Work income				
Yes	1 (2%)	25 (20%)	*x*^2^ = 7.6 p = 0.006**	26 (15%)
Sex work				
Yes	21 (48%)	0	*x*^2^ = 69.6 p < 0.001***	21 (12%)
Theft				
Yes	15 (34%)	50 (39%)	*x*^2^ = 0.3 p = 0.557	65 (38%)
Dealing				
Yes	14 (32%)	50 (39%)	*x*^2^ = 0.7 p = 0.391	64 (37%)
Monthly income > 33,000 NKR^b^				
Yes	27 (61%)	57 (45%)	*x*^2^ = 3.7 p = 0.054*	84 (49%)
Years of injecting career				
> 5 years	32 (73%)	105 (82%)	*x*^2^ = 1.2 p = 0.279	137 (80%)
**Drug use in the previous four weeks**				
Daily or almost daily injections				
Yes	42 (95%)	113 (88%)	*x*^2^ = 1.9 p = 0.169	155 (90%)
Heroin most injected				
Yes	43 (98%)	113 (88%)	*x*^2^ = 3.5 p = 0.063*	156 (91%)
To have injected > 12.9 grams of heroin^c^				
Yes	29 (66%)	67 (52%)	*x*^2^ = 2.4 p = 0.118	96 (56%)
Combined heroin and prescription drugs in injections				
Yes	20 (45%)	33 (26%)	*x*^2^ = 5.9 p = 0.015**	53 (31%)
Any use of prescription drugs				
Yes	32 (73%)	67 (52%)	*x*^2^ = 5.6 p = 0.018**	99 (58%)
Alcohol ≥2 days a week or more				
Yes	10 (23%)	31 (24%)	*x*^2^ = 0.04 p = 0.841	41 (24%)
Cannabis ≥2 days a week or more				
Yes	13 (30%)	52 (41%)	*x*^2^ = 1.7 p = 0.191	65 (38%)

*p < 0.10, **p < 0.05, ***p < 0.001.

^a^In Norway all children are expected by law to attend school for 10 years. Prior to 1997, it was nine years.

^b^The cut-off was set at the median total income in Norwegian Kroner in the total sample. In 2013 33,000 NKR amounts to 3,560 GBP.

^c^The cut-off was set at the median amount of heroin injected by the total sample which was 12.9 grams of heroin injected in the past month.

Table 1 also shows that the majority of participants (91%) had injected mainly heroin rather than amphetamine and other opioids, in the four weeks prior to inclusion. A higher proportion of the women injected mainly heroin compared to the men (98% vs. 88% *x*^2^ = 3.5, p = 0.063). Yet there were no significant gender differences in those who had used more heroin than the median amount used by the total sample (>12.9 grams). However, more women combined prescription drugs in heroin injections (45% vs. 26% *x*^2^ = 5.9, p = 0.015). Further, more women used prescription drugs in the month previous to the study, than men (73% vs. 52% *x*^2^ = 5.6, p = 0.018).

### Mortality and assessment of risk factors

The cohort was followed for a total of 1,927 PY. By 2010, 45 participants died; 8 females and 37 males. Women had a lower CMR than the men (16.0 [95% CI 8.0, 31.9] vs. 26.0 [95% CI 18.0, 35.8]), but these differences were not statistically significant. Conversely, men had a lower SMR than the women (21.3 [95% CI 5.7, 54.1] vs. 39.4 [95% CI 0.2, 220.8]).

**Figure 1:**
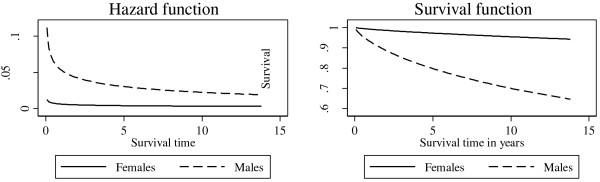
Hazard and survival function* by gender using Log-Logistic distribution. *Adjusted for age, length of injection career, combination of prescription drugs in heroin injections, alcohol use and prostitution as a source of income.

The highest number of deaths occurred in the first two years of follow-up. Eight participants died in the first year of the study and nine participants died in the second year. Of those who died, women died on average (median) 1.2 years after the baseline interview and only one female died after three years. The deceased men died on average (median) 5.1 years after the baseline interview and 23 died after three years. Figure 1 shows that men had a higher hazard function than the women throughout the study period. However, both men and women had a high hazard function in the first two years and thereafter a decrease. The survival function in Figure 1 illustrates that women had higher probability of survival than men.

Table 2 shows that when survival for men and women were analysed using both unadjusted and adjusted log logistic regression there were no significant gender differences (Table 2). Survival time significantly decreased on a 10% significance level (p = 0.055) when participants had combined heroin and prescription drugs in injections in the previous four weeks before the interview (TR = 0.4 [95% CI 0.01, 1.0]. When accounting for unobserved heterogeneity (Model 2), the estimates remained similar to the model without (Model 1) and, the likelihood-ratio test was not statistically significant (*x*^2^ = 0 p = 1.000). This means that unobserved individual effects were negligible in this model.

**Table 2:** Unadjusted and adjusted time ratio (TR) for survival time using Log-Logistic regression analysis, with and without unobserved heterogeneity (“frailty”)

	**Model 1: total population**	**Model 2: total population**		**Model 3: women**^**a**^	**Model 4: men**^**a**^
		**No frailty**^**a**^	**Frailty**^**b**^		
	**Unadjusted**	**Adjusted**	**Adjusted**	**Adjusted**	**Adjusted**
	**TR**^**c **^**[95% CI]**	**TR**^**c **^**[95% CI]**	**TR**^**c **^**[95% CI]**	**TR**^**c **^**[95% CI]**	**TR**^**c **^**[95% CI]**
Male	0.5 [0.2, 1.7]	0.5 [0.1, 1.7]	0.5 [0.1, 1.7]	-	
≥ 30 years^d^	0.8 [0.3, 2.1]	0.8 [0.3, 2.3]	0.8 [0.3, 2.3]	0.1 [0.0, 4.5]	1.3 [0.5, 3.6]
> 5 years IV career^e^	0.8 [0.2, 2.7]	1.1 [0.3, 3.8]	1.1 [0.3, 3.8]	1.0 [0.0, 152.3]	1.1 [0.3, 3.7]
Combined heroin and prescription drugs^f, g^	0.4 [0.2, 1.1]*	0.4 [0.1, 1.0]*	0.4 [0.1, 1.0]*	0.2 [0.0, 5.3]	0.5 [0.2, 1.2]
Alcohol ≥2 days a week^g^	0.8 [0.3, 2.3]	0.8 [0.3, 2.4]	0.8 [0.3, 2.4]	0.3 [0.0, 11.8]	0.8 [0.3, 2.3]
Sex work^g^	0.4 [0.1, 1.7]	-	-	0.02 [0.00, 1.8]*	-
n	171	171	171	43	128
Gamma	-	1.4 [1.0, 1.8]	1.4 [1.0, 1.8]	1.8 [1.0, 3.4]	1.2 [0.9, 1.6]
Theta	-	-	0	-	-
Log-Likelihood	-	-161.7	-161.7	-30.7	-125.2
Akaike’s Info. Crit.	-	337.5	339.5	75.4	70.7
Likelihood-ratio test of theta = 0	-	-	0.0 p = 1.000	-	-

*p < 0.10.

^a^Without unobserved heterogeneity.

^b^With unobserved heterogeneity.

^c^Time ratio for covariate *k =* exp (β*_k_).

^d^At inclusion.

^e^> 5 years between first injection and interview.

^f^Combined heroin and prescription drugs in injections.

^g^In the previous four weeks before the baseline interview.

Since no men reported sex work as an income source, it could not be included in the full model. Instead women were analysed separately. Women who reported income from sex work had 98% decreased survival time (TR = 0.02 [95% CI 0.0, 1.8]) and this was significant on a 10% significant level (p = 0.090).

In the post hoc analysis, we assessed the differences in baseline characteristics between women who reported income from sex work (n = 21) and those (n = 23) who did not. There was a higher proportion “older than 30 years” among those who reported sex work as an income source (67% vs. 35%, p = 0.069). Fewer of those in sex work reported income from theft (19% vs. 48%, p = 0.060). Those in sex work reported a higher mean monthly income (57,600 NKR vs. 38,200 NKR, p = 0.006). Most importantly, a higher proportion of those in sex work used prescription drugs in combination with heroin the previous four weeks (90% vs. 57%, p = 0.017). Additionally, although not statistically significant, women who reported income from sex work had used slightly more prescription drugs in addition to heroin in the day before the interview (9.4 tablets vs. 6.0 tablets, p = 0.178); slightly more heroin per injection (0.18 g vs. 0.16 g, p = 0.445); used heroin for slightly more days in the past month (29.0 days vs. 27.8 days, p = 0.512); and consequently consumed slightly more heroin in total in the past month (23.5 g vs. 21.4 g, p = 0.715).

As described in the Methods section, information on substitution treatment, incarceration and prison release was only available between 1.1.1998 and 31.12.2004. With this limited data set the study population was 169, as three people died before 1.1.1998 (1 female and 2 males). In this smaller data set there was a significant difference between the proportion of men and women who had been in prison during the study period. Of 43 women, 51% had been in prison compared to 74% of the 126 men (*x*^2^ = 7.6, p = 0.006). On the other hand, there was no significant difference between the proportion of women and men who had been in substitution treatment (44% vs. 41% *x*^2^ = 0.1, p = 0.738).

Table 3 shows the effects of OST, incarceration and prison release on the risk of mortality using cox regression survival analysis. This table shows that for each year in OST, the risk of death was reduced by 30% (HR = 0.7 [95% CI 0.05, 0.9]) and for each year in prison, the risk was reduced by 90% (HR = 0.1 [95% CI 0.0, 0.6]). Prison release (<3 weeks) increased the risk of mortality more than four times (HR = 4.3 [95% CI 0.5, 34.7]), however this was not significant, not even on a 10% significance level (p = 0.169) when also adjusting for covariates (HR = 3.4 [95% 0.7, 20.4]). For women, each year in OST reduced the mortality risk by 99% (HR = 0.1 [95% CI 0.0, 0.6]) and each year in prison reduced the mortality risk by 97% (HR = 0.03 [95% CI 0.0, 0.7]) also in the adjusted model. In this adjusted model sex work increased the mortality risk almost twenty-three times (HR = 22.7 [95% CI 1.5, 33.8]). It was not possible to estimate the effect of prison release on women due to the small sample size.

**Table 3:** **Adjusted effects of substitution treatment, incarceration and prison release on risk of mortality assessed using Cox regression analysis in a limited study period 1.1.1998 and 31.12.2004 (n = 169)**^**ab**^

	**Model 1: total population**	**Model 2: total population**	**Model 3: women**	**Model 4: men**
	**Adjusted HR**^**b **^**[95% CI]**	**Adjusted HR**^**b **^**[95% CI]**	**Adjusted HR**^**b **^**[95% CI]**	**Adjusted HR**^**b **^**[95% CI]**
Male	-	1.7 [0.7, 4.3]	-	-
≥ 30 years^c^	-	0.8 [0.3, 1.9]	1.5 [0.2, 12.1]	0.5 [0.2, 1.3]
> 5 years IV career^c, d^	-	1.5 [0.5, 4.2]	0.7 [0.0, 13.8]	1.7 [0.5, 5.2]
Heroin and prescription drugs^e, f^	-	1.4 [0.7, 3.0]	0.8 [0.1, 4.1]	1.5 [0.6, 3.5]
Alcohol ≥2 days a week^f^	-	1.1 [0.5, 2.3]	2.6 [0.3, 20.8]	1.1 [0.5, 2.5]
Sex work^f^	-	-	22.7 [1.5, 333.8]**	-
Total years in OST	0.7 [0.5, 0.9]**	0.7 [0.5, 0.9]**	0.1 [0.0, 0.6]**	0.7 [0.5, 1.0]**
Total years in prison	0.1 [0.0, 0.6]***	0.1 [0.0, 0.6]***	0.0 [0.0, 0.7]**	0.1 [0.0, 0.6]**
Prison release^a^	4.3 [0.5, 34.7]	3.7 [0.4, 30.3]	-^g^	3.2 [0.6, 16.4]

*p < 0.10, **p < 0.05, ***p < 0.001.

^a^Data was only available from OST from 1.1.1998 when OST was established as a national programme in Norway. The follow-up time for this table is therefore from 1.1.1998-31.12.2004. Three cases died before 1.1.1998 and therefore had to be omitted; the study population was therefore 169 and not 172 as in the original sample.

^b^It was not possible to run the log logistic regression model due data organization. Please see the Methods section for more detail. However, this model satisfied the proportionality assumption and therefore a Cox regression survival analysis could be used. In this model HR and 95% CI are reported.

^c^At inclusion.

^d^> 5 years between first injection and interview.

^e^Combined heroin and prescription drugs in injections.

^f^In the previous four weeks before the baseline interview.

^g^Unable to estimate the effect of prison release on women due to a small sample size.

### Causes of death

Table 4 shows the causes of death in detail. Of the 45 who died, 60% died from acute intoxications (25 cases) and dependence syndrome (2 cases). Therefore, acute intoxications were the most common cause of death. Seventeen (5 females and 12 men) of the acute intoxications were due to the use of opioids. Seven (all men) were defined as accidental poisoning by and exposure to narcotics and psychodysleptics. One male death was classified as acute intoxication due to use of sedatives or hypnotics. The other causes of death are found in Table 4.

**Table 4:** Primary causes of death of the 45 deaths recorded during follow-up

**Primary causes of death**	**Women n = 8 (100%)**	**Men = 37 (100%)**	**Total n = 45 (100%)**
Acute intoxications			
Due to the use of opioids	5 (63%)	12 (32%)	17 (38%)
Due to use of sedatives or hypnotics	0	1 (3%)	1 (2%)
Accidental poisoning by and exposure to narcotics and psychodysleptics [hallucinogens], not elsewhere classified	0	7 (19%)	7 (16%)
Dependence syndrome	1 (12%)	1 (3%)	2 (4%)
Suicide	1 (12%)	2 (5%)	3 (7%)
Acute infections (meningococcal infections and sepsis)	0	2 (5%)	2 (4%)
Chronic infections (hepatitis C and HIV)	0	4 (11%)	4 (9%)
Other causes (traffic accidents, drowning, asthma, malignant neoplasm of other connective and soft tissue)	1 (12%)	8 (22%)	9 (20%)

## Discussion

The risk of mortality was highest in the first two years after inclusion for both genders. Within the first three years, 22 of the 172 participants died (13%). The deceased females died median 1.2 years and males 5.1 years after inclusion. This suggests that women were more at risk in the short-term, but more protected in the long-term. The most common cause of death was acute intoxications with no significant gender differences. The risk factors associated with decreased survival time were combining prescription drugs in heroin injections and sex work (only women in this study). In sub-analyses conducted on a smaller data set, prison release did not significantly increase the mortality risk, while time in prison significantly decreased the risk. A significantly higher proportion of men had been in prison. The same proportion of men and women had been in OST and the mortality risk was reduced in both genders while they were in treatment. Therefore, the two gender specific risk factors found in this study were sex work and prison.

Women appeared to be more vulnerable in the short-term as the majority of the women who died, died in the first three years after inclusion. Women had a more hazardous pattern of substance use in the weeks before study inclusion. More women had injected heroin and they had consumed similar amounts of heroin as the males and with the same frequency. Further, female IDUs consumed more prescription drugs and more frequently combined prescription drugs with heroin injecting. They also used alcohol as frequently as the men. Injection frequency, heroin injections, use of prescription drugs and alcohol use are all factors that are known to increase the risk of premature mortality among drug users [9,13,48]. The pattern of substance use among the female participants is likely to be one of the reasons why the majority of the women who died, died in the first three years after the interview. However, the combination of prescription drugs in heroin injections was not statistically significant when women were analysed separately. Possibly in a larger female population it would have been possible to detect a significant association between the pattern of substance use and death.

Despite the few female deaths, sex work was significantly associated with survival time on a 10% significance level and this is similar to the findings from a Canadian study [27]. It could be that sex work decreased survival time due to risk of HIV and HIV-related causes of death. However, none of the women in our study died from HIV-related causes. Instead, six of the females died from acute intoxications, one from suicide and one from other causes. It is not likely that sex work increases the risk of acute intoxications per se. However, sex work is likely to be an indicator of hazardous behaviour that directly increases the risk. There might be unobserved commonalities among those who reported sex work as an income source, which in turn are associated with survival time. It was not possible to assess for heterogeneity when women were analysed separately due to the small sample size and the question in regards to unobserved heterogeneity among women therefore remains unanswered.

On the other hand, it could be that the high income from sex work allowed these women to consume more drugs and thereby shortening their survival time. To explore this further, the women who reported income from sex work were compared to women who did not. This analysis indicated that a higher proportion of those from the former group had a higher total monthly income and a more hazardous drug use than those from the latter group. The association between sex work and mortality should be researched further to understand if there is a causal link between the two.

Combining different substances such as prescription drugs and heroin is a well-known risk factor for acute intoxications [13,50,53]. One study found that for every additional drug used, the odds ratio for mortality almost doubled [12]. In our study, survival time for those who combined prescription drugs in heroin injections decreased by 60%. Based upon previous literature [12,13,50,53] and the findings from this study, it is likely that combination of different substances is directly associated with shorter survival time.

The survival function in Figure 1 shows that women had a much higher probability of survival than men. Only one female died after the first three years of follow-up, whereas 23 men died in the same time period. Women were therefore more protected in the long-term. One possible reason for the higher probability of survival could be that female drug users sought treatment earlier in their drug career and they were less likely to relapse [28,29,54]. Treatment reduces the risk of mortality [55-57]. Possibly treatment was more accessible to women than men during the study period and thereby increasing females’ probability of survival. Furthermore, women are less likely to be incarcerated [24,25,37] and less likely to re-offend [38,39]. It could be that gender differences in incarceration and treatment increased females’ probability of survival.

To assess the effect of substitution treatment and prison episodes on the mortality risk, we re-analysed the data using a smaller data set. As hypothesized, fewer females had been incarcerated and this could indeed be one reason why more females survived than men. Yet contrary to our initial hypothesis that release from prison significantly increased the mortality risk [35,37,58], the estimates in this study were not significant not even on a 10% significance level. Instead, each year in prison significantly decreased the mortality risk by 30%. This means that to spend time in prison was protective, while prison release did not significantly increase the risk. More men, than women, were incarcerated during the study period. Viewed in isolation it would seem men were more protected. Yet overall more men died. This suggests that there must be other gender specific risk factors not observed in this study, that increase the risk more for men than for women. These unobserved gender specific risk factors should be investigated in future studies to discover how to improve survival among male drug users.

It was found that time in OST significantly decreased the risk of death which is in accordance with previous literature [55-57,59]. A priori we hypothesized that more females would be in OST compared to males, and thereby be more protected. Contrary to what was expected it was the same proportion of females and males who had been in OST during the study period (44% vs. 41%). This means that OST was not a gender specific factor that protected women due to a higher proportion of women in OST. However, we did not have information regarding other types of treatment and it may be that more women than men, had access to alternative treatment.

The majority of deaths occurred in the first three years of follow up. A rapid increase of OST patients, in particular after the year 2000 [43], could possibly be one reason why there were fewer deaths after three years. On the other hand, it may also be that those who died in the early years of follow-up were those who were particularly vulnerable, while those who had survived up until three years after inclusion had characteristics that enhanced survival. Though we did not explore this in our study. There could also be other factors not identified in this study that protected the participants after three years. It is likely that the reasons why fewer died after three years are due to a combination of the reasons listed above.

### Limitations and strengths

One of the study’s limitations is sample size which limits the statistical analyses in regards to possibilities of findings statistical significance at a 5% level. Additionally, common to all studies that use self-reported data, weaknesses of our study might include selection bias, recall bias, under and over-reporting, and imprecise estimation of illegal activities. Although the quality of the National Cause of Death Registry is deemed reliable for the date of deaths, the primary cause of deaths, which depends on assessment by individual doctors, is somewhat more vulnerable for misclassifications [60].

Another limitation was that the respondents were not asked in detail about amphetamine and other opioid use. This information would have given a more detailed picture of the respondents’ substance use at the time of the interview.

Our study did not include information about those who refused to participate in the baseline interview and so it is not known how these people differed from those who participated, if at all. However, the gender and age distribution of our sample was similar to what was observed for IDUs in Norway at the time [44], which should suggest that they did not differ. Regardless, this is not known and the lack of this information may have biased the results in some manner.

One of the main strengths of our study is that it was a prospective longitudinal cohort study that followed the study participants over 13 years. This enabled us to compare short- and long-term survival between men and women. Despite the small sample size it was possible to detect significant differences at a 5% significance level. We also obtained data from those who refused to participate in the long-term cohort study and a comparison showed that the participants did not differ from those who did not participate. This reduced the chances for selection bias. Finally, participants were street-recruited which was unique as these people may not be available for inclusion in treatment or prison-based studies. The reason for this is that they may never have accessed treatment or been to prison. This study gives insights into a population that are currently exposed to the risks factors related to injecting drug use.

## Conclusion

This study found gender differences in mortality and in the risk factors for premature death, which may be useful in developing preventive measures. Women were more vulnerable in the short-term, whilst more protected in the long-term. One of the gender specific risk factors found in this study was sex work, associated only with females. Therefore, female IDUs who use sex work as an income source should therefore be addressed specifically. It may be assumed that male IDUs would experience the same level of risk associated with sex work. However the findings from our study could not confirm this assumption. Contrary to our initial hypothesis, OST was not a gender specific risk factor. The same proportion of men and women had been in OST during the study period and the mortality risk was significantly reduced in both genders whilst they were in treatment. Prison was a gender specific risk factor, as a significantly higher proportion of men had been in prison. Time in prison significantly decreased the mortality risk, while prison release did not significantly influence the risk. As such, it was expected that men should be more protected than women, yet they were not. Therefore there must be other factors causing an increase in mortality risk more among men, but these factors were not uncovered in this study. Improved understanding of these gender differences could help to reduce the mortality risk in both male and female IDUs. Finally, the combining of different substances should be a significant consideration in developing preventive measures, as this was associated with a shorter survival time, for both men and women.

## Competing interests

There are no competing interests to report.

## Authors’ contributions

ALBJ designed the study, wrote the research protocol and carried out the data collection. LG analysed and interpreted the data, undertook the literature searches and summaries of previous related work and drafted the manuscript. ALBJ participated in the interpretation of the data and revised the manuscript critically for intellectual content. Both authors read and approved the final manuscript.

## Pre-publication history

The pre-publication history for this paper can be accessed here:

http://www.biomedcentral.com/1471-2458/14/440/prepub
